# Therapeutic Effect of Seaweed Derived Xanthophyl Carotenoid on Obesity Management; Overview of the Last Decade

**DOI:** 10.3390/ijms21072502

**Published:** 2020-04-03

**Authors:** Oyindamola Vivian Ojulari, Seul Gi Lee, Ju-Ock Nam

**Affiliations:** 1 Department of Food Science and Biotechnology, Kyungpook National University, Daegu 41566, Korea; hoyeendahmolar@gmail.com (O.V.O.); lsg100479@naver.com (S.G.L.); 2 Institute of Agricultural Science and Technology, Kyungpook National University, Daegu 41566, Korea

**Keywords:** carotenoids, seaweeds, antioxidants, astaxanthin, fucoxanthin, anti-obesity, oxidative stress

## Abstract

Present-day lifestyles associated with high calorie-fat intake and accumulation, as well as energy imbalance, have led to the development of obesity and its comorbidities, which have emerged as some of the major health issues globally. To combat the disease, many studies have reported the anti-obesity effects of natural compounds in foods, with some advantages over chemical treatments. Carotenoids, such as xanthophyll derived from seaweeds, have attracted the attention of researchers due to their notable biological activities, which are associated mainly with their antioxidant properties. Their involvement in oxidative stress modulation, the regulation of major transcription factors and enzymes, and their antagonistic effects on various obesity parameters have been examined in both in vitro and in vivo studies. The present review is a collation of published research over the last decade on the antioxidant properties of seaweed xanthophyll carotenoids, with a focus on fucoxanthin and astaxanthin and their mechanisms of action in obesity prevention and treatment.

## 1. Introduction

Obesity—defined as the excessive or abnormal accumulation of body fat in the adipose tissue, energy imbalance, and lipogenesis—results from modern lifestyles characterized by high intakes of fat, sugar, and calories, in addition to poor exercise and physical activity [1,2]. It is a global epidemic with over 2.1 billion cases, accounting for about 5% of all global deaths [3] and has been projected to rise drastically, especially in the adult population, in the near future [4,5]. The societal impact of obesity has been categorized to include huge personal, social, and economic costs [3,6]. A report stated that obese people are susceptible to depression and emotional trauma from discrimination, lower wages, and a lower quality of life [3]. Additionally, the medical costs incurred from the diagnosis and treatment of obesity and associated health conditions such as heart disease, high blood pressure, diabetes, stroke, etc. have been estimated to be between 2%–7% of health-care expenses (about $2.0 trillion) in developed economies [6].

The molecular mechanism of obesity mediated by cytokines, adiponectin, and leptin has been correlated with increasing inflammation and oxidative stress, and leads to the development of metabolic diseases including certain types of cancer; hyperglycemia; type 2 diabetes; high blood pressure; and liver, heart, and gallbladder diseases [2,7]; which pose major health risks globally [8]. Specifically, obesity and type 2 diabetes are associated with an increase in oxidative stress and inflammation in adipose tissues [2,9,10]. Consequently, researchers have been exploring functional materials of plant origin that contain antioxidants to combat obesity and its comorbidities, as alternatives to conventional approaches that have been adopted previously—including surgery and anti-obesity drugs, which do not often have enduring effects and have side effects including headaches, excessive thirst, insomnia, constipation, and steatorrhea [11], or may have yo-yo effects [8,12]. Therefore, the consideration of antioxidants as therapeutic agents in the treatment and/or prevention of obesity and its comorbidities would be insightful.

Seaweed generally refers to plants and algae that grow in waterways such as oceans, lakes, rivers, and streams. They can be classified based on their pigmentation as brown algae, red algae, and green algae [13,14], and are natural sources of bioactive compounds such as polyphenols, lipids, and carotenoids, which exhibit antioxidant activities and other beneficial properties [15], with broad applications in the food, feed, and pharmaceutical industries [16]. In some Asian countries such as Korea and Japan, seaweeds are widely consumed as functional foods for their health benefits, and are major traditional food items (e.g., Kimbap) [17,18]. The prevalence of various metabolic syndromes in this region is reportedly lower than in western countries such as the USA and Australia [14], suggesting that dietary habits influence human health. Seaweeds contain carotenoids, which function as antioxidant, anti-viral, anti-cancer, anti-obesity, anti-inflammatory, anti-coagulant, and anti-lipemic agents [19]. Some bioactive compounds that have been isolated and identified from seaweeds include carotenoid pigments such as fucoxanthin (FXN) [20], astaxanthin (ASX), zeaxanthin, and β-carotene [21,22].

Carotenoids are the largest group of lipid-soluble natural pigments, and they are responsible for the red, orange, and yellow colors in many fruits and vegetables [23,24]. Fruits and vegetables containing carotenoids have been reported to be antioxidant-rich and suitable for the formulation of functional foods to be consumed for their health benefits [25] In addition, they are associated with reduced risks of chronic diseases [26]. The dietary intake of carotenoid-containing vegetables such as seaweeds could reduce the risks of the development of cardiovascular disease, age-related macular degeneration, obesity, and certain cancer types [27,28,29,30,31,32], and is linked with a longer life expectancy and a lesser risk of metabolic diseases [33]. They have also been reported to exhibit antioxidant properties including radical scavenging ability and the quenching of singlet oxygen, and to perform biological and physiological functions, [34] and—coupled with other processes—to have positive impacts on human health [35]. Moreover, animals and humans are unable to synthesis carotenoids, hence the need for direct supplementation. Furthermore, some of these carotenoid pigments (including α-carotene and β-carotene) are provitamin A, which perform the single function of metabolic conversion to vitamin A (retinol) [36]. However, ASX and FXN are non-provitamin A xanthophylls [37,38].

Carotenoids are divided into carotenes and xanthophylls (e.g., FXN and ASX). Both xanthophylls and carotenes are potent antioxidants; however, xanthophylls have been reported to exhibit higher antioxidant activity, and various beneficial impacts on human physiology [39]. Xanthophylls are oxygen-containing carotenoids, and are relatively abundant pigments found in the chloroplasts of seaweed [40] In seaweeds, FXN and ASX are the major xanthophyll carotenoids that exhibit high antioxidant activity due to their singlet oxygen quenching and free radical scavenging abilities [17]. Numerous studies have investigated the antioxidant potential of the carotenoids and their metabolites with regard to their role in obesity management [30]. A recent study reported that xanthophyll intake could improve lipid metabolism and reduce abdominal fat and Body Mass Index (BMI) in healthy overweight individuals [41]. Over the past decade, a new perspective on the biological function of seaweed carotenoids and their potential applications in the treatment of obesity and obesity-related diseases has emerged [35,42]. Some studies have reported the therapeutic effects of the compounds in adipose tissue biology to include adipogenesis (adipocyte differentiation), the modulation of adipocyte metabolism, and oxidative stress regulation, in addition to the modulation of the expression of specific adipokines and pro-inflammatory factors [43,44,45,46]. The mechanism of action of carotenoids has been attributed to their ability to prevent adipocyte hypertrophy and adipogenesis while enhancing the oxidation of fat and energy recreation in brown and white adipocytes, which, in turn, controls obesity in animal models [30,47].

The objective of the present review is to collate the findings of studies conducted over the past decade on the antioxidant properties of seaweed carotenoids, with a focus on xanthophylls (FXN and ASX), including findings on their relevance to obesity treatment and the mechanism of action by which they achieve the functions in vitro (in cells), in vivo (in animal models), and in clinical studies (in human subjects).

## 2. Antioxidant Properties and Bioaccessibility of Seaweed Xanthophyll Carotenoids

One of the main biological properties of seaweed carotenoids is their antioxidant properties. Their antioxidative potentials are beneficial as defensive mechanisms against cellular inflammation [2,9,48] and oxidative stress [2,9,48], and play major roles in the prevention and treatment of obesity and obesity-related diseases [7,8,10]. When consumed in appreciable amounts, edible seaweeds supply the body with potent antioxidants and enhance its ability to counteract excessive oxidative stress in the body [34]. Various seaweeds species have been demonstrated to exhibit good antioxidant effects and suppress oxidative stress in vitro and in vivo [49]. Studies have estimated the antioxidant properties of extracts and isolates from various seaweed species using different in vitro assays such as 2,2-Diphenyl-1-picrylhydrazyl (DPPH), ferric reducing antioxidant capacity (FRAP), or oxygen radical absorbance capacity (ORAC) and 2,2′-Azino-bis (3-ethylbenzothiazoline-6-sulfonic acid (ABTS), among others. [16,40,50,51,52,53,54,55,56,57,58]. In addition, the active compounds in seaweeds, such as sulphated polysaccharides, phlorotannins, and—particularly—carotenoids, have been suggested to be the major antioxidant compounds responsible for the beneficial health effects, and a lot of research has focused on the potential applications of the bioactive compounds in obesity management [59,60].

Both FXN and ASX are accumulated in the pigments of different seaweed species [38,61] While FXN is peculiar to brown-algae (e.g., *Undaria pinnatifida*), ASX is found in substantial amounts in green-algae (e.g., *Haematococcus pluvialis*). In their single compounds, these carotenoids (FXN and ASX) are extracted from seaweeds by organic solvents such as acetone, chloroform, ethanol, diethyl ether, etc., accompanied by simple extraction techniques including maceration [62,63] and vortex microextraction [64], as well as innovative extraction technologies such as pressurized liquid [65], supercritical fluids [66,67,68,69], microwave extraction [70,71], ultrasound extraction [72,73], and enzyme-assisted [74,75] extraction. The FXN (orange-pigmented) and ASX (red-pigmented) xanthophyll carotenoids from seaweed have been reported to exhibit high antioxidant capacities [76]. Seaweed (*F. vesiculosus*) extracts containing 0.0012% FXN, showed high antioxidant activity (DPPH, ABTS, and ABAP) and inactivated RAW 264.7 macrophages [20,61]. Similarly, the consumption of FXN and ASX carotenoids has been associated with reduced levels of oxidative stress-mediated inflammation in relation to obesity [43]. Over the past decade, accumulating evidence has demonstrated a link between the carotenoids and adipocyte/adipose tissue biology [42]. A recent study also revealed that the intake of xanthophylls improves lipid metabolism and reduces abdominal fat and BMI in healthy overweight individuals [41].

Naturally, xanthophyll carotenoids are present as esters (90% of marine-derived xanthophylls), which is essential to their bioavailability [77]. Unlike carotenes, which are known to be converted into Vitamin A, the accumulation and bioavailability of FXN and ASX vary among animal and human models [37,77]. The accumulation of dietary FXN and ASX (either intact or as metabolites) in the tissues have been reported in animal models [78,79,80,81], but few recent studies are available on bioavailability in humans [82,83]. Hence, it is inexpedient to draw a relative conclusion on the quantity of xanthophylls available for absorption in animal and human models. Regardless, marine xanthophylls have good bio-accessibility [77]. Metabolic mechanisms such as gut-hydrolysis, deacetylation, and the oxidative conversion of xanthophylls to their respective metabolites have been identified in animal models for the absorption of xanthophylls and their metabolites in the tissues [78,79,80,81]. However, more studies are required to unravel the metabolism and mechanism of absorption in humans, identifying the key enzymes and molecular mechanisms involved in the metabolic conversion of xanthophylls. Additionally, the bioavailability of carotenoids in seaweeds may largely be influenced by factors including the food matrix, the processing methods, cooking, and the structures of carotenoids [37] when consumed as food.

## 3. Fucoxanthin and Obesity

**Figure 1:**
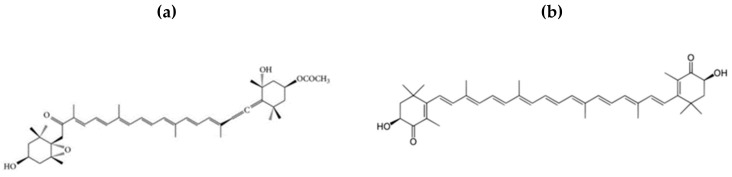
The chemical structures of (**a**) fucoxanthin and (**b**) astaxanthin.

FXN (Figure 1a), a seaweed carotenoid, has gained popularity recently due to its antioxidant properties, and is therefore considered a protective agent that could decrease the oxidative-inflammatory status associated with body weight gain, and it is being applied in the treatment of the various diseases triggered by obesity [84,85]. FXN is present in the chloroplasts of brown seaweed such as *Hizikia fusiforme, Fucus serratus, Laminaria, Alaria crassifolia, japMiyatonica, Fucus vesiculosus, Sargassum horneri,* and *Undaria pinnatifida* and has been isolated for its bioactivity [61,86,87,88,89]. The unique characteristics of FXN lie in its structure (containing an allenic bond), which accounts for its distinctive therapeutic effects [44,89,90]. Health-related, dietary FXN has gained substantial awareness due to its beneficial effects in humans. Also, fucoxanthin was reported to possess the most effective anticarcinogenic activity among xanthophylls; thus, fucoxanthin and other seaweed carotenoids have therapeutic potentials and could be used as nutraceuticals, functional food ingredients, and alternatives for synthetic antioxidants [91].

### 3.1. Therapeutic Potential of Fucoxanthin in Obesity

FXN isolated from *Pinnafida binghamiae* reportedly exerts anti-obesity effects in 3T3-L1 adipocytes by inhibiting the differentiation of adipocytes at both intermediate and late stages, in addition to inhibiting glucose uptake in mature adipocytes [92]. Various effects of FXN on obesity parameters have been observed with regard to body weight, visceral fat, adipose tissue size, fasting blood glucose concentrations, plasma insulin levels, and lipid and cholesterol metabolism rates, among others (Table 1). Reductions in bodyweight gain, white adipose tissue (WAT) weight, visceral fat, hepatic total lipids, total cholesterols, and plasma and liver triglyceride concentrations are usually accompanied by increased fecal lipid excretion, which has been observed and reported in various in vivo studies of diet-induced obesity [44,93,94,95,96]. Based on these studies, FXN supplementation has been effective in enhancing the expression of lipid-metabolizing enzymes such as carnitine palmitoyltransferase-1 (CPT1) and Cholesterol 7α-hydroxylase1 (CYP7A1) [46], promoting fatty acid oxidation [45,62], and suppressing the activity of cholesterol-regulating enzymes such as 3-hydroxy-3-methylglutaryl coenzyme A reductase and acyl-coenzyme A [44,96], and these effects have been used to explain the aforementioned actions. Dietary fucoxanthin supplementation in KK-A^y^ mice increased serum cholesterol levels, attenuated fat accumulation, and suppressed the enlargement of visceral WAT during the development of obesity [84]. However, there are inconsistent reports with regard to the effective FXN dosages.

A low FXN supplementation (10% lard + 10% corn oil + 0.05% FXN *w*/*w*) in the diet of 5-week old C57BL/6N mice fed for 6 weeks was reported to lower plasma triglyceride and liver lipid concentrations [44]. Surprisingly, similar effects were also observed in the same mouse model, fed with more FXN (10% lard + 10% corn oil + 0.2% FXN *w*/*w* supplementation) [44]. In another study, 4-week old male Sprague-Dawley rats fed with a high-fat diet (HFD) and 0.2% FXN (13% lard + 7% soybean oil + 0.2% FXN *w*/*w*) for 4 weeks had significant decreases in hepatic total lipid, total cholesterol, and triglyceride concentrations compared to the HFD group [46]. Airanthi et al. [20], reported a decrease in lipid hydroperoxide levels in the liver and abdominal WAT weight in 5-week old female KK-A^y^ mice fed for 4 weeks with a diet supplemented with seaweed lipids (11.51% soybean oil + 2% lipid extracts) containing FXN 16–21 mg/g lipid. Meanwhile, in a 16-week clinical study involving non-diabetic, obese premenopausal female humans, a minimum FXN dose of 2.4 mg/day significantly promoted weight loss and body and fat content reduction and enhanced liver functions in obese female humans with an average weight of approximately 100 kg [45]. Also, FXN administration (3 mg/day) for 4 weeks in 50 male and female adults (1:1, aged 20–59 years) reduced body weight, BMI, and abdominal fat in the obese patients [95]. The effect of FXN on anti-obesity parameters seems similar at different concentrations. However, the amount or dosage of FXN required to exert certain anti-obesity effects could vary depending on study models (human or animals), due to varying sensitivity [40] or different absorption rates [89]. Therefore, further clinical studies are necessary for a more unanimous report on its effective dosage. Furthermore, as a co-administered drug, the synergetic effects of FXN in combination with other compounds or materials in the treatment of obesity have been investigated [45,96]. A combination of FXN and linoleic acid decreased body weight gain and improved lipid metabolism in HFD-induced obese rats [96]. Similarly, combination with pomegranate seed oil reduced body weight, liver fat, and triglyceride concentrations significantly in obese premenopausal women [45]. Also, the direct consumption of wakame seaweed (*Undaria pinnatifida*)—which is rich in carotenoids, particularly FXN—has been reported to effectively reduce postprandial glucose and insulin responses in healthy adults within 30 min of consumption with rice [105]. In fact, cooked wakame has a higher FXN concentration than fresh samples [106], due to improved bioavailability upon cooking [37]. With regard to toxicity, most seaweeds are edible, and thus FXN isolated from seaweeds could be considered as a safe pharmaceutical ingredient [107]. Furthermore, a clinical trial reported no abnormalities or adverse effects exhibited by individuals who ingested FXN [95].

### 3.2. Mechanisms of the Anti-Obesity Effect of Fucoxanthin

Several studies in the literature have attributed the anti-obesity mechanism of fucoxanthin to the modulation of key transcriptional factors related to obesity. Among these, the nuclear receptor PPARγ and C/EBPα are master regulators of adipogenesis [108,109], playing a crucial role in the differentiation and function of mature adipocytes [110] The activation of these nuclear receptors in adipocytes has been reported to enhance insulin resistance associated with obesity [109,111]. PPARγ, when expressed in appreciable amounts in fat tissues, significantly induce adipogenesis [108,110]. Both PPARγ and C/EBPα bind most induced genes linked to adipogenesis and metabolism [112], implying a coactive upregulation of adipogenic gene expression by these two key regulators [100]. These nuclear receptors (PPARγ and C/EBPα) positively regulate each other’s expression [113], and cooperate to promote their respective stimulated adipogeneses (C/EBPα ↔PPARγ-stimulated adipogenesis) [109,114].

A study by Kang et al. [92] revealed FXN to present anti-obesogenic effects on 3T3-L1 adipocyte cells during the three differentiation (early, middle, and late) stages. The promotion of adipocyte differentiation and the increased protein expression of PPARγ, CCAAT/enhancer-binding protein α (C/EBPα), sterol regulatory element-binding protein 1c (SREBP1c), and adiponectin mRNA expression occurred at the early stage, while intercellular lipid accumulation was restrained by a reduction in the expressions of transcriptional factors (PPARγ, C/EBPα, and SREBP1c) during the intermediate and late differentiation stages of adipocyte cells. This study related the anti-obesity and anti-adipogenic effects of FXN to its structural characteristics, which promote the downregulation of the expression of key regulatory proteins including PPARγ, C/EBPα, and SREBP1c. Further investigation confirmed that FXN treatment increased the phosphorylation of adenosine monophosphate-activated protein kinase (AMPK) and acetyl-CoA carboxylase (ACC), as well as liver kinase B1 (LKB1) phosphorylation, and decreased the expression of SREBP1c in mature 3T3-L1 adipocytes. SREBPs (including SREBP-1a, SREBP-1c, and SREBP-2) are other key transcriptional factors similarly involved in cholesterol homeostasis, and are induced during the differentiation of preadipocytes [113]. SREBP-1c regulates fatty acid and cholesterol synthesis, providing lipid ligands that mediate PPAR activation and promote adipogenesis [108]. FXN enhanced the cholesterol synthetic pathway in the liver—by increasing the transcriptional factors SREBP2 and SREBP1, and the mRNA expression levels of 3-hydroxy-3-methyl-glutaryl-coenzymeA reductase (HMGCR), 3-hydroxy-3-methyl-glutaryl-coenzymeA synthase (HMGCS), farnesyl diphosphate Synthase (FDPS), and cytochrome P450 14α-sterol demethylase (CYP51) enzyme involved in cholesterol synthesis—which decreased hepatic cholesterol content in the FXN fed mice compared with in the control mice [84]. Moreover, the scavenger receptor B type 1 (SR-B1) and low-density lipoprotein receptor LDLR proteins (which play an important role in modulating circulating cholesterol levels in the liver) were also observed to be downregulated by FXN, and these proteins result in increased serum cholesterol levels and the reduced hepatic clearance of serum cholesterol [84].

Conversely, the findings in in vivo studies suggest that the administration of *Pinnafida binghamiae* extracts (containing FXN150 mg/kg/day) inhibited high-fat-diet (HFD)-induced obesity by increasing fatty acid β-oxidation and inhibiting de novo lipogenesis in the adipose tissue of C57BL/6 obese mice [98]. The downregulation of stearoyl-coenzyme A desaturase-1 (SCD1), and the enhancement of insulin and leptin sensitivity have been suggested as a curative measures in HFD-induced obesity [97,102,105]. Leptin, predominantly produced in adipose cells, regulates body and fat weight by regulating food intake and energy expenditure. A comparative in vivo study using hyperleptinemia KK-Ay and leptin-deficiency ob/ob mouse models revealed that FXN reduced body weight gain, visceral WAT mass, and serum leptin levels by downregulating hepatic SCD1 expression through the regulation of leptin signaling in KK-Ay mice with hyperleptinemia [97]. However, the effects were not remarkable in leptin-deficient ob/ob mice, suggesting the mechanism of action of FXN to be by the downregulation of SCD1 expression through the regulation of leptin signaling.

Another study attributed the action of FXN on insulin resistance in HFD-induced KK-Ay diabetic obese and C57BL/6J lean mice to the regulation of the mRNA expression of inflammatory adipocytokines (iNOS and COX-2) in the WAT of diabetic/obese KK-Ay mice [85]. Cyclooxygenases (COX) are important regulators of metabolism [115]. At the mRNA level, the expression of cyclooxygenase-1 (COX-1) is induced in adipocytes, and cyclooxygenase-2 (COX-2) expression is induced in inguinal-WAT (iWAT) with regards to the induction of UCP 1 [115,116,117]. The overexpression of COX-2 was accompanied by decreased iWAT mass and adipocytes, improved insulin-stimulated glucose disposal, and reduced markers involved in hepatic steatosis in HFD C57BL/6 mice [115]. Besides, UCP1 expression in WAT is modulated by the expressions and activities of PPARα, PPARγ [98], and COX-2 [98,116,117], which have been reported to be altered by fucoxanthin [1,44,98].

The anti-obesity mechanism of fucoxanthin has been linked to the upregulation of uncoupling protein 1 (UCP1), which is a key molecule for metabolic thermogenesis to avoid an excess of fat accumulation, and acts as a physiological defense against the onset of obesity [1,59,91,118]. Generally, UCP1 is a notable component of energy expenditure in the body that is expressed in the brown adipose tissue (BAT) [91], mediates the oxidation of fatty acids, and promotes energy expenditure by thermogenesis. FXN induces (UCP1) in abdominal WAT, mediates the oxidation of fatty acids and heat production, and promotes energy expenditure by thermogenesis. Correspondingly, the anti-obesity impact of FXN may be linked to the browning of white adipocytes through the upregulation of UCP1, which yields increments in energy expenditure in the body [118].

## 4. Astaxanthin and Obesity

ASX (Figure 1b), a red-pigmented xanthophyll carotenoid, exhibits antioxidant activity that is approximately 10 times more potent than that of any other carotenoid, including α-carotene and β-carotene [119,120], and that is about 100 fold that of α-tocopherol [38,121]. Its reactive oxygen scavenging ability and anti-inflammatory properties have been studied in both in vitro and in vivo [122,123], and they exhibit protective effects against metabolic diseases including cardiovascular diseases and obesity [99,101]. Studies have shown that the unique chemical structure (the presence of polar moieties on both ends of its polyene chain) of ASX greatly influences its antioxidant potency and ability to remove free radicals and excessive reactive oxygen species [124]. Besides possessing antioxidant activities, ASX was found to have antagonistic effects on adipocytes and agonistic effects on peritoneal macrophages, acting as a selective PPARγ modulator, related to obesity [119].

ASX showed health-promoting potential, such as considerable preventive and curative abilities against oxidative stress-related diseases including obesity [107]. It was suggested that ASX prevented HFD-induced obesity by lowering plasma triglycerides, total cholesterol levels, body weight, and adipose tissue size. ASX has also shown potential in preventing mitochondrial damage, and the alleviation of oxidative stress associated with nonalcoholic fatty liver disease [108]. Thus, AXN is regarded as a promising therapeutic agent that is effective and safe for obesity management. [109].

### 4.1. Therapeutic Potential of Astaxanthin in Obesity

Oxidative stress is a major underlying cause for metabolic disorders and is characterized by insulin resistance, which is related to obesity. Insulin is an important hormone maintaining glucose homeostasis [125]. In obese mice, fat cells were observed to be more resistant to insulin, causing the accumulation of glucose in the blood [102]. Moreover, ASX exerts beneficial effects on glucose and lipid metabolism in diet-induced animal models, by lowering insulin resistance (according to the homeostasis index of insulin resistance) and decreasing fat accumulation [126]. ASX treatment was also reported to ameliorate insulin resistance and improve insulin signaling by activating post-receptor insulin signaling [85,112], to reduce the oxidative stress produced by various stimuli including TNF-α [111], and to inhibit pro-inflammatory cytokines in obese mice [85,110]. ASX improved the blood lipid profile by decreasing markers of lipid peroxidation, reducing triglyceride and low-density lipoprotein-cholesterol (LDL-C) levels, and increasing high-density lipoprotein cholesterol (HDL-C) [94,100], and equally exhibited anti-obesity activity and inhibitory effects on adipogenesis in mouse models [26,92]. In vivo studies showed the administration of ASX to significantly reduce the body weight and adipose tissue weight gain induced by a high-fat diet, and to also reduce liver weight, liver triglycerides, plasma triglycerides, and total cholesterol by stimulating an increase in fatty acid utilization [125].

Choi et al. [103] reported that a 3-week ASX (5 and 20 mg/day) supplementation in human suppressed lipid peroxidation and improved oxidative stress biomarkers by stimulating the antioxidant defense system. A dose-dependent decrease in plasma malondialdehyde and isoprostane levels, accompanied by increases in plasma total antioxidant capacity and superoxide dismutase levels in overweight and obese young adults, was observed in the study [103]. In addition, improved antioxidant defense mechanisms and improved lipid metabolism following ASX supplementation have been reported in mouse models [100,101]. ASX supplementation (0·03%) in HFD-fed apoE knockout (apoE)−/− mice reduced plasma cholesterol and triglyceride levels; however, there was no significant change in body weight when compared with the control group [101]. The observed hypocholesterolemic effect was attributed to an increase in the mRNA expression of the low-density lipoprotein receptor and in fatty acid β-oxidation in the livers of ASX-fed mice. In their further studies, the authors investigated the effect of ASX on lowering plasma triacylglycerol concentrations [100]. According to their results, feeding C57BL/6J mice with 0.03% ASX similarly induced significantly lower plasma triacylglycerol concentrations, with a significant increase in the fatty acid synthase and diglyceride acyltransferase 2 mRNA abundances than in the control mice. They concluded that ASX could prevent obesity-associated metabolic disturbances and inflammation by increasing the hepatic expression of endogenous antioxidant genes, and rendering splenocytes less sensitive to lipopolysaccharide stimulation.

A clinical study by Yoshida et al. [99] investigated the effects of ASX on the lipid profile in humans. ASX concentrations of 12–18 mg/day reduced triglyceride concentrations significantly, which were correlated with increases in serum adiponectin hormone concentration, while 6–12-mg/day doses increased high-density lipoprotein cholesterol significantly. However, BMI remained unaltered at all doses [99]. The increased adiponectin concentrations following ASX consumption were suggested to be due to its anti-inflammatory properties; however, this was not demonstrated in the study.

### 4.2. Mechanisms of the Anti-Obesity Effect of Astaxanthin

Adiponectin is an abundantly expressed adipose-specific adipokine that produces insulin-sensitizing effects, and is directly involved in regulating glucose levels and fatty acid metabolism [127]. In obese subjects, the levels of adiponectin are usually low, which is associated with lower degrees of insulin sensitivity and glucose tolerance, and a higher adipose TNF-α expression [128,129,130]. Leptin is another important adipokine abundantly expressed in adipose tissue and is involved in the balancing of energy homeostasis [127]. Leptin helps to control food intake, energy expenditure, and, hence, body weight [128].

From this point of view, the anti-obesity mechanism of ASX might be mediated by improving adipokine levels (i.e., reducing leptin levels and increasing adiponectin levels). In fact, ASX-mediated adiponectin elevation has been observed in human trials [99]. Since adiponectin is regulated by the peroxisome proliferator-activated receptor-γ (PPARγ) pathway and is involved in close-inhibition relationships with inflammatory cytokines [99], the dysregulation of the nuclear receptor peroxisome proliferator-activated receptor δ/β (PPARδ/β) could be associated with obesity, because of its key role in lipid metabolism [131]. Arunkumar et al. [102] attributed the improvement in insulin resistance, reduced lipid levels, and adipose tissue weight observed in obese mice to the PPARγ agonist and modulation effects of ASX supplementation. Furthermore, ASX, upon direct binding with PPARγ, induced adipogenesis in 3T3-L1 cells by inhibiting rosiglitazone (a PPARγ ligand) and PPARγ transcriptional activity [47,119]. Clearly, the integrated activation of PPARδ/β (connecting the muscle and adipose tissues) by ASX could have led to the induction of non-shivering thermogenesis, in turn reducing fat accumulation in adipocytes and controlling body fat reduction [131].

In obesity-induced insulin resistance, inflammatory cytokines and free fatty acids (FFAs) released from adipose tissue are involved in insulin sensitivity [132], and are reportedly caused by abnormal insulin signaling in obese models [133]. High glucose and fatty acid levels associated with obesity lead to increased reactive oxygen species (ROS) production, which causes insulin resistance in metabolic tissues [134]. The beneficial effect of ASX on ROS-induced insulin resistance in obese mouse models has been linked to the modulation of signaling pathways, primarily mitogen-activated protein kinase (MAPK) and JNK (c-Jun N-terminal kinase) pathways [102,133]. Since collated evidence confirms the roles of cytokines such as TNF-α in the promotion of insulin resistance by the generation of ROS or the activation of JNK in inflammatory pathway signaling, the inhibition of JNK could be an important target for the treatment of inflammation-induced insulin resistance or abnormal glucose tolerance in obesity [133,134]. As a potent antioxidant, ASX reportedly improved insulin resistance through the JNK-IRS-1-Akt axis by suppressing insulin-induced JNK phosphorylation and IRS-1 serine phosphorylation in the liver of HFD mouse [134,135]. Also, ASX significantly decreased ROS produced by oxidants including TNF-α and enhanced Akt phosphorylation related to the increase in GLUT4 translocation or glucose uptake [126].

The expression of nuclear factor erythroid 2-related factor 2 (NRF-2), a responsive antioxidant gene, is important in the cellular defense against oxidative stress. The transcription of key antioxidant genes is induced by NFR-2, thereby decreasing the concentrations of oxidative stress markers, such as glutathione disulfide (GSSG), in the livers of mice [100]. ASX has been able to enhance the antioxidant defense mechanisms and prevent non-alcoholic fatty liver disease (NAFLD) by improving lipid metabolism in HFD-fed C57BL/6J and apoE 2/2 mice [100,101]. The mechanism was further explained to be due to the induction of NRF-2, which caused a significant 2-fold increase in the mRNA expression of NRF-2; moreover an increase in the expression of targeted endogenous antioxidant genes (superoxide dismutase 1, glutamate–cysteine ligase regulatory subunit, and glutathione peroxidase 1), was evenly observed in the liver of ASX-fed mice [100,124]. Some recent studies have substantiated that NRF-2 inducers improve insulin resistance in obesity models and increase glucose uptake by decreasing oxidative stress in the hypothalamus [136,137].

Additionally, Yang et al., [100] reported the riacylglycerol (TAG) lowering effects of ASX as observed in ASX supplemented mice, which was attributed to the expression of genes involved in lipogenesis and fatty acid β-oxidation. The increase in the mRNA expression of acyl-CoA oxidase 1 (ACOX-1) and increases in the hepatic expression of 3-hydroxy-3-methylglutaryl-CoA reductase (HMGR), the LDL receptor, and the rate-limiting enzyme in peroxisomal fatty acid β-oxidation were observed in ASX- fed mice; however, no difference was observed in the expression of SREBP-2 [100,101]. 

Overall, ASX has been proven by various studies as a beneficial natural antioxidant that improves insulin resistance and lipid metabolism and that ameliorates oxidative stress-induced obesity. Moreover, the ingestion of ASX is considered safe [38,39,122,138].

## 5. Conclusions

**Figure 2:**
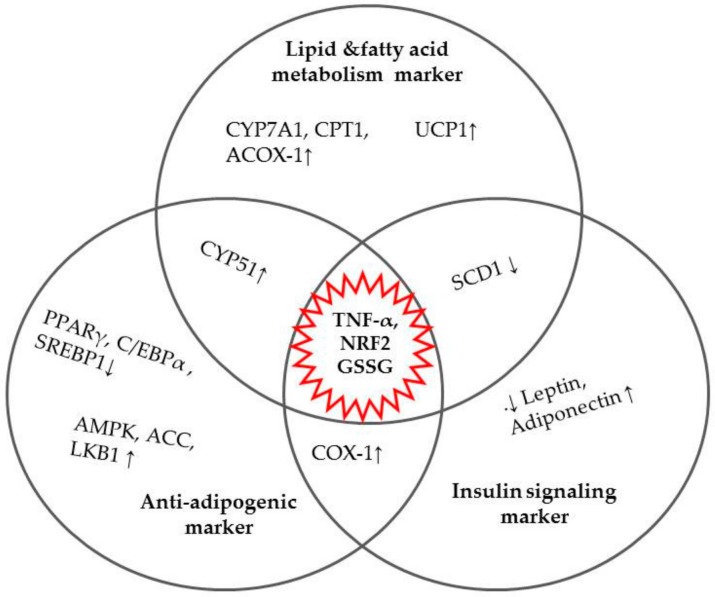
Transcription factors, mRNA expressions, and cytokines involved in the anti-obesity mechanism of the fucoxanthin (FXN), and astaxanthin (ASX) xanthophyll carotenoids. Arrow pointing down (↓) represents down-regulation, while upward position (↑) denoted up-regulation.

The prevalence of obesity is of global concern, with the accumulated evidence tending towards oxidative stress-induced maladies, making the supplementation of natural antioxidant a feasible intervention. This review presented the relationships between antioxidants, oxidative stress, inflammation, and obesity, with a clear illustration that seaweed carotenoids are antioxidant-packed bioactive compounds with potent oxidative defense mechanisms and anti-obesity properties. Xanthophyll carotenoids (FXN and ASX), as described in the reviewed studies, positively modulated the expressions of various transcriptional factors, cytokines, and enzymes associated with obesity, substantiating their therapeutic potentials for obesity management. The molecular mechanisms of action on various obesity parameters have been highlighted in this review (Figure 2). Moreover, these xanthophyll carotenoids have proven to positively mediate the abnormalities caused by excess ROS and the depletion of antioxidant system in obese subjects. Thus, a detailed understanding of their mechanisms could evolve new ideas in the treatment of obesity and peripheral metabolic diseases. Based on the safety and molecular mechanisms highlighted, FXN and ASX showed good pharmacological effects on obesity and could be potential materials for functional foods and drugs for human health benefits. However, since the availability and metabolic fate of carotenoids vary between animal and human models, it is inexpedient to conclude regarding the effective supplementation doses of FXN and ASX in the treatment of obesity.

## Figures and Tables

**Table 1:** Reported studies on the effects of selected xanthophylls and their anti-obesity effects.

s/n	Subject/Model	Effective Dose	Seaweed Specie	Observed Effect	References
1	Male Sprague Dawley Rat	0.083 and 0.167 mg/kg/bw FXN supplementation in HFD	not mentioned	Plasma and liver triglyceride concentrations were reduced and cholesterol-regulating enzymes such as 3-hydroxy-3-methylglutarylcoenzyme A reductase and acyl-coenzyme A were positively influenced.	[96]
2	Obese KK-Ay mice	0.2% FXN diet	*Undaria pinnatifida*	The increase in HDL and non-HDL (High-Density Lipoprotein) cholesterol levels; reduction in liver uptake of cholesterol were observed in KK-Ay mice.	[84]
3	KK-Ay mice and B6. V-Lepob/J (ob/ob) mice	0.1 or 0.2% FXN supplemented diet	*Undaria pinnatifida*	Suppressed body weight, visceral WAT mass, and lowered serum leptin levels.	[97]
4	Mice	150 mg/kg/day	*Petalonia binghamiae*	PBE (or FXN) exert improving effects on HFD-induced obesity by promoting β-oxidation and reducing lipogenesis.	[98]
5	C57BL/6J and KK-Ay mice	0.2% fucoxanthin FXN	*Undaria pinnatifida*	FXN regulated mRNA expression of inflammatory adipocytokines, and attenuated both body weight gain and WAT weight in diabetic/obese KK-Ay mice, but did not affect these parameters in lean C57BL/6J mice	[85]
6	Obese female volunteers with 100 kg average weight	2.4 mg/kg/day	*Undaria pinnatifida*	Increased energy expenditure in the body and resulted in significant weight loss after 16 weeks.	[45]
7	Female KK-Ay mice	2% seaweed lipids containing FXN 16–21 mg/g	*Undaria pinnatifida (Wakame), Sargassum horneri (Akamoku), and Cystoseira hakodatensis (Uganomoku)*	Significant decrease in liver lipid hydroperoxide levels and abdominal WAT weight.	[20]
8	C57BL/6N mice	0.05% and 0.2% FXN in diet, *w*/*w*	*Undaria pinnatifida*	FXN supplementation improves plasma and hepatic lipid metabolism and blood glucose concentration.	[44]
9	C57BL/6J mice	0.02% dose	*Undaria pinnatifida*	Ethanol extract on diet-induced-insulin resistance in C57BL/6J mice	[94]
10	C57BL/6J mice	0.05 or 0.2% FXN supplemented diet	*Undaria pinnatifida*	Regulated plasma and hepatic lipid metabolism; increased fecal lipid excretion. Fecal weight, liver, and triglycerides and cholesterol were not significantly different between 0.05 and 0.2% FX fed mice.	[62]
11	30 male 4-week-old C57BL/6 mice	Oral; 150 mg/kg/day for 70 days	*Petalonia binghamiae*	Extract (PBE) administration reduced body weight gain, adipose tissue weight, adipose cell size, serum triglyceride levels, and reduced lipid accumulation in the liver in HFD-induced obese mice.	[98]
12	3T3-L1 adipocyte	10 µM treatment	*Petalonia binghamiae*	FXN activated the AMPK signaling pathway; increased the phosphorylation of LKB1, AMPK, and ACC; and inhibited the expression of PPARγ, C/EBPα, and SREBP1c in mature 3T3-L1 adipocytes.	[92,98]
13	Male Sprague-Dawley rats	0.2% FXN powder in feed	*Undaria pinnatifida*	The levels of hepatic lipids cholesterols and triglycerides were reduced significantly, with subsequent increases in the fecal excretions of lipids, cholesterol, and triglycerides in the FXN supplemented group.	[46]
14	Sprague-Dawley rats	5% seaweeds powder supplemented in HFD	*Eucheuma cottonii; aulerpa lentillifera, and Sargassum polycystum*	All seaweeds significantly reduced body weight gain and plasma lipid peroxidation in HCF diet rats. However, *S. polycystum* showed the best anti-obesity properties.	[49]
15	KK-Ay mice and B6. V-Lepob/J (ob/ob) mice	0.1 or 0.2% FXN supplemented diet	*Undaria pinnatifida*	Suppressed body weight, visceral WAT mass, and lowered serum leptin levels.	[97]
16	C57BL/6N mice	0.05 or 0.2% FXN supplemented diet	*Undaria pinnatifida*	Regulated plasma and hepatic lipid metabolism; increased fecal lipid excretion. Fecal weight, liver, and triglycerides and cholesterol were not significantly different between 0.05 and 0.2% FX fed mice.	[62]
17	Human	6, 12, and 18 mg/day of ASX	*Haematococcus pluvialis*	12 and 18 mg/day improved the serum lipid profile in humans (decrease in TG levels observed), 6 and 12 mg/day increased HDL-cholesterol; however, BMI values remained unchanged.	[99]
18	Male C57BL/6J mice	HF diet supplemented 0.003, 0.01 and 0.03% of ASTX (by weight)	*Hematococcus pluvialis*	0.03% ASX fed group showed significantly lowered triacylglycerol concentrations; increased the hepatic expression of endogenous antioxidant genes.	[100]
19	Male apoE knockout (apoE)−/− mic	0.03% ASX	*Haematococcus pluvialis*	ASX-rich H. pluvialis extract improves cholesterol and lipid metabolism as well as antioxidant defense mechanisms.	[101]
20	Male Swiss albino mice	6 mg/kg per day in olive oil for 60 days	*Haematococcus pluvialis*	ASX treatment reduced lipid accumulation and oxidative stress and adipose tissue weight. Also improved insulin sensitivity.	[102]
21	Human; overweight and obese young adults	5 mg and 20 mg soft ASX capsule	not mentioned	Improved lipid metabolism and prevented oxidative stress by stimulating the activity of the antioxidant defense system.	[103]
22	Male mus musculus albino mice	6 mg/kg per day in olive oil for 60 days	*Haematococcus pluvialis*	ASX improved antioxidant status, restricted weight gain, enhanced insulin sensitivity and restored liver lipid levels.	[104]

## Abbreviations

ABTS	2,2′-Azino-bis (3-ethylbenzothiazoline-6-sulfonic acid
DPPH	2,2-Diphenyl-1-picrylhydrazyl
ABAP	2,2′-azobis(2-amidopropane)
ACC	Acetyl-CoA carboxylase
AMPK	AMP-activated protein kinase
ASX	Astaxanthin
BMI	Body mass index
TNF-α	Tumor necrosis factor α
C/EBPα	CCAAT Enhancer Binding Protein Alpha
COX-2	Cyclooxygenase-2,
FRAP	Ferric reducing antioxidant capacity
FXN	Fucoxanthin
iNOS	Inducible nitric oxide synthase
mRNA	Messenger RNA
ORAC	Oxygen radical absorbance capacity
PPARγ	Peroxisome Proliferator-activated Receptor γ
SCD1	Stearoyl-coenzyme A desaturase-1
SREBP-1c	Sterol regulatory element-binding protein
WAT	White adipose tissue
